# Significance of concentration-dependent viscosity on the dynamics of tangent hyperbolic nanofluid subject to motile microorganisms over a non-linear stretching surface

**DOI:** 10.1038/s41598-022-16601-9

**Published:** 2022-07-27

**Authors:** Imran Siddique, Sohaib Abdal, Irfan Saif Ud Din, Jan Awrejcewicz, Witold Pawłowski, Sajjad Hussain

**Affiliations:** 1 Department of Mathematics, University of Management and Technology, Lahore, 54770 Pakistan; 2 Department of Mathematics, Khwaja Fareed University of Engineering and Information Technology, Rahim Yar Khan, Pakistan; 3 School of Mathematics, Northwest University, No. 229 North Taibai Avenue, Xi’an, 7100069 China; 4 Department of Automation, Biomechanics and Mechatronics, Lodz University of Technology, 1/15 Stefanowskiego St., 90-924 Lodz, Poland; 5 Institute of Machine Tools and Production Engineering, Lodz University of Technology, Lodz, Poland; 6 School of Aerospace and Mechanical Engineering, Nanyang Technological University, Singapore, Singapore

**Keywords:** Engineering, Mathematics and computing

## Abstract

The communication describes a theoretical framework for tangent hyperbolic fluid of nano-biofilm due to an extending or shrinking sheet that comprises a stagnation point flow, chemical reaction with activation energy, and bioconvection of gyrotactic microorganisms. The varying transport features due to dynamic viscosity, thermal conductivity, nano-particle mass permeability and microbe organisms diffusivity are taken into account for the novelty of this work. The inspiration is developed to enhance heat transfer. A set of leading partial differential equations is formed along with appropriate boundary constraints. Using similarity transformations, the basic formulation is transitioned into non-linear differential equations. To produce observational data, the shooting technique and Runge-Kutta fourth order method are employed. The coding of numerical scheme is developed in Matlab script. The visual representation of the effects of diverse fluid transport properties and distinctive parameters on speed, temperature, concentration and motile density are evaluated. The velocity become faster when the parameters $\omega$, $\lambda$, $\epsilon$ and $V_0$ are enhanced. Brownian motion, thermal conductivity, heat generation as well as thermophoresis factors all strengthen the temperature distribution, however the nano-particle concentration profile is enhanced as the nano-particle mass conductivity variable, activation energy as well as the thermophoresis variable are boosted. The microorganism density improves significantly when the microorganism diffusivity factor increases. The skin friction, Sherwood number, Nusselt number and motile density number decline against the incremented transport parameters.

## Introduction

The tangential hyperbolic fluid is a non-Newtonian fluid that is used in nuclear engineering systems. It offers certain benefits over other non-Newtonian fluids. Blood, sauces, paints, nail varnish, condensed milk are samples of tangent hyperbolic fluid. Non-Newtonian fluids achieve a nonlinear connection with effective stress and elastic deformation related to their complicated chemical composition and are hence classified as power law models. The tangent hyperbolic method produces shear rate results, i.e., viscosity decreased as shear rate increased. Kumar et al.^1^ examined the effect of a magnetic field on Casson nano liquid movement over a curved stretching/shrinking sheet with chemical reaction. Khan et al.^2^ probed the generation of entropy in Darcy–Forchheimer hybrid nanofluid flow over a stretchable surface using Marangoni convection. Kumar et al.^3^ used the KKL model to investigate the effect of a magnetic dipole on the radiative flow of nanofluid over a stretching and shrinking sheet. Gowda et al.^4^ evaluated the Stefan Blowing Effect on the Flow of Second-Grade Fluid Over a Curved Stretching/Shrinking Sheet using computational methods. Gowda et al.^5^ reviewed the deposition of thermophoretic particles in the flow of a hybrid nanofluid suspension by ferrite nanoparticles through an expansion/contraction rotating disc. Jamshed et al.^6^ used a single-phase mathematical model to investigate the radiation heat transfer of a second-grade nanofluid flowing past a porous flat surface. Ibrahim and Gizewu^7^ used the bvp4c approach to analyze the influence of nonlinear mixed convection flow of a hyperbolic tangent fluid using the Cattaneo-Christov mass and heat diffusion system through a bidirectional stretch sheet with activation energy.

Khan et al.^8^ examined the circulation, heat, and mass transmission characterization of a chemically resistant mixed convective stream of hyperbolic tangent liquid in a doubly stratification medium. Kumar et al.^9^ scrutinized the contribution of Arrhenius activation energy inside the stream and heat transport of tangent hyperbolic fluid having zero mass flux situation, which was handled numerically using the RKF-45 approach. Shafiq et al.^10^ utilized a shooting approach to examine the barrier layer flow (BLF) along a vertical gradually extending surface with a composite of mass and thermal transmission ratio in tangent hyperbolic nanofluid including microbes. Ullah et al.^11^ employed Lie group analysis to scrutinize the Magneto-hydrodynamic tangent hyperbolic liquid motion across an extending sheet with suction/injection impact at the boundary.

The distribution of nanoparticles in common fluids (water, oil, ethylene glycol, and so on) to improve their thermophysical properties has made this a fascinating research issue. Nanoparticles are nanometer-sized particles made of metals and/or metal oxides that have the ability to significantly increase the thermal energy carrying capacity of ordinary fluids. The nanocomposite combinations are chosen with the goal of incorporating both nanoparticles, valuable properties into a single balanced, homogenous structure. Aly et al.^12^ explored the role of a magnetic field on the thermosolutal convection of solid particles in a finned cavity containing solid particles. Abdal et al.^13^ explored the role of activation energy and different transit variables in the two-dimensional stagnation-point flow movement of a nano-biofilm of Sutterby fluids containing gyrotactic microbes across a highly permeable straining/shrinking sheet. Habib et al.^14^ looked into the magnetic effects of heat and mass transfer on the flow of a micropolar fluid via a porous stretching geometry with a dilute homogeneous presence of nanoparticles and gyrotactic microorganisms. Habib et al.^15^ examined the multi-slide effects of time-dependent MHD nanofluid transpiration in the presence of a magnetic and electric field on a stretching sheet containing suspended rising live creatures. Shi et al.^16^ studied the magnetised flow of cross nano liquid past a stretched sheet that was subjected to heat radiation.

Microorganisms are single cells that exist in all living things, including animals, humans and flora. Microorganisms create gyrotactic and they are significantly thicker than water due to the accumulation of microbes. The physical and intriguing importance of gyrotactic microorganisms is effectively used in bioenergy, ethanol, and other environmental and ecological systems. Bioconvection is a method in which limited microbes float near the surface of the fluid due to unstructured circumstances and volatility. Swimming microbes, such as microalgae, have the ability to grow across the upper liquid layer, which is responsible for the unstable higher section, leading to a higher density of stratified. Bioconvection is used in a variety of applications, including biomedical applications and bio micro systems, pharmaceutical companies, biological polymer synthesis, environmentally conscious applications, self-sustaining fuel cell techniques, microorganism petroleum refining, biomaterials and biotech and continuous numerical method refinement. Kotha et al.^17^ examined the two-dimensional magneto-hydrodynamic stream and mass and heat transmission features of water-based nanoparticles comprising gyrotactic microbes across a vertical plate utilizing heat production or absorbance. Alshomrani et al.^18^ scrutinized the movement of a non-Newtonian magnetic cross nanoparticles with mass and heat transfer ratios, activation energy, motile microbes and bioconvection across a wedge using the bvp4c technique. Abbasi et al.^19^ proposed the circulation of a viscoelastic nanofluid containing gyrotactic microorganisms across a revolving extending disc having a convective boundary as well as zero mass diffusion situations by applying the Keller Box approach. Yusuf et al.^20^ investigated the rate of entropy generation in a bio-convective flow of a magnetohydrodynamic Williamson nanoliquid over an inclined convectively heated stretchy plate, taking into account the effects of heat radiation, permeable materials, and chemical reaction. Kakarantzas et al.^21^ researched natural convection of liquid metal MHD in a vertical cylindrical container with a sinusoidal temperature profile at the upper wall and other surfaces. Benos and Sarris.^22^ examined the two-dimensional flow pattern and steady-state MHD natural convection of a nanofluid-filled shallow cavity with internal thermal generation. Benos et al.^23^ reviewed laminar two-dimensional natural convection in a shallow cavity filled with an aqueous carbon nanotube (CNT) nanofluid and subjected to internal heating and an external constant magnetic field. Waqas et al.^24^, Ferdows et al.^25^ and Rao et al.^26^ deliberated the motion of gyrotactic microbes in distinct aspects.

The area of stagnation point is critical in flow characteristics in industrial and natural processes. All solid amounts dissolved in liquids exhibit the basic presence of a stationary point. Because of its wide range of uses in both commercial and scientific settings, the stagnation point flow has piqued the interest of many academics. Product development and manufacturing, extrusion operations, plane counter-jets and other kinds of hydraulic modeling in engineering are some of the practical applications of stagnation point flow. Khan and Alzahrani^27^ explored the influence of Brownian motion and thermophoresis as well as heat and mass transmission behaviors on the nonlinear heat radiating stagnation point movement of a Walter-B nanofluid. Anuar et al.^28^ reported analytical results for homogeneous-heterogeneous magneto-hydrodynamic (MHD) stagnation point motion of ${\text{Cu}}{-}{\text{Al}}_2{\text{O}}_3$/water hybrid nanoparticles generated by an expanding or contracting sheet having a convective boundary constraint. Zainal et al.^29^ utilized the bvp4c algorithm to evaluate the unstable three-dimensional magneto-hydrodynamic non-axisymmetric Homann stagnating point stream of a hybrid ${\text{Al}}_2{\text{O}}_3{-}{\text{Cu}}/{\text{H}}_2{\text{O}}$ nanofluid. Nadeem et al.^30^ addressed the stable three-dimensional stagnation stream affected by a permeable movable system with anisotropic slip as well as a magnetic flux inside the fluid domain and heat dispersion. Gul et al.^31^ demonstrated the significant effects of the magnetic field upon the 2D, time-dependent and stagnant point incompressible viscous motion of a pair of stressed hybrid nanofluids along a revolving sphere, having the base fluid being fresh blood as well as the nanoparticles having ${\text{TiO}}_2$ and *Ag* by employing the methodology of Optimal Homotopy Analysis.

In a significant variety of nuclear and thermal-hydraulic operations, heat transport, including fluid movement, is required. A range of fluids and operational conditions have been explored in an attempt to improve the heat transfer mechanism. The function of cooling is critical in maintaining the required thermal performance in a variety of engineering and technical goods, such as laptops, motor vehicles, chemical processes, laptops and strip conditioning, drying operations, thermal collectors, hydroelectric extraction, hydropower breaks, heating systems, cooling of microprocessors, food manufacturing, glass production, heating elements and solar thermal energy are a few examples. Refiei et al.^32^ studied the solar-driven organic Rankine cycle (ORC) scheme with a point of focus magnifier and 2 distinct cavity-shape recipients as the ORC heat origin from thermodynamics, financial and environmental perspective. Tayebi et al.^33^ scrutinized the heat transmission features of a ${\text{Cu}}{-}{\text{Al}}_2{\text{O}}_3/{\text{H}}_2{\text{O}}$ premised hybrid nanofluid filled annulus designated by two elliptical cylinders incorporating organic convection, movement as well as entropy formation. Aziz and Shams^34^ utilized a shooting strategy in accordance with the fourth order RK approach to examine the volumetric entropy generation frequency in an electrically arranged Maxwell nanofluid across a climbable elongating sheet with varying heat conductivity, velocity slip situations, thermal radiation and inner heat origin influence. Armaghani et al.^35^ presented a computational assessment of entropy creation owing to MHD-free convection of Cu-water nanoparticles in a permeable I-shaped cavity using the finite difference approach. Shi et al.^16^ studied the transient magnetic flow of cross nanoliquid past a stretching sheet using radiant heat, binary chemical reactions, thermal source effects, and convective boundary conditions. Kumar et al.^36^ explores the nature of the Arrhenius activation energy in the flow of hybrid nanoparticles of manganese zinc ferrite (MnZnFe_2_O_4_) and nickel zinc ferrite $({\text{NiZnFe}}_2{\text{O}}_4)$ with Kerosene oil as a base liquid over a curved stretchable surface (CSS) in the presence of exponential heat generation. Li et al.^37^ explored the entropy nature of a steady, laminar, and relative contributions of thermal and solutal Marangoni convections on passage in a Casson Al2O3 Cu H2O hybrid nanofluid stream past over a disc under the influence of a nonlinear heat source/sink, viscous dissipation, radiation, and nonlinear convection. Xiong et al.^38^ studied the two-dimensional Darcy-Forchheimer flow of various hybrid nanofluids under the impact of a consistent heat source sink and non-linear thermal radiation. Wang et al .^39^ researched the heat and mass transport phenomena in the 3D flow of Oldroyd-B fluid subjected to the Soret and Dufour effects with radiant heat and magnetic force. Other researchers^40–43^ discussed the varied implementations of heat sources.

In the disciplines of oil resource engineering and thermal reservoirs, activation energy is also essential. The phrase activation energy is essential in chemical reactions. In reality, it is the lowest amount of energy required to transform the reactants into materials. Activation energy can take the form of kinetic or stored energy. Svante Arrhenius, a Swedish physicist, coined the phrase activation energy for the first time in 1889. The activation energy, indicated by *Ea* and quantified in KJ/mol, indicates the atoms or molecules’ minimum energy required to begin the chemical process. Khan et al.^44^ investigated the rheology of a pair stress nanofluid incorporating activation energy, permeable media, heat flux, gyrotactic microbes, as well as convection Nield boundary situations. Muhammad et al.^45^ assessed a mathematical evaluation for three-dimensional Eyring-Powell nanofluid non-linearity heat radiation across a Riga plate containing updated thermal plus mass oscillations and slip boundary constraints. Khan et al.^46^ explored the magneto-hydrodynamic mashed convection 2nd grade nanofluid stream across a permeable medium in the existence of heat radiation, heat absorption/generation, buoyancy impacts and entropy formation as well as the Arrhenius activation energies and bipolar chemical change. Shah et al.^47^ presented the thermal transfer assessment through joule dissipation, thermodynamic properties as well as convective boundary constraint of a radiative electrically charged Casson nanofluid across a nonlinearly expanding sheet using entropy production. Gowda et al.^48^ explored the role of binary chemical change and activation energy on a nanofluid’s steady Marangoni-driven boundary layer flow and heat and mass transfer characteristics^49–51^ scrutinized the influences of activation energy on distinct nanofluids motions.

When going through the related studies, it seems that concentration dependent properties of tangent hyperbolic nanofluid are rarely investigated. The nano-particle diffusion changes the nature of base fluid to be described as non-Newtonian fluid regime. Moreover, the probable agglomeration of nano-entities can be avoided in presence of bioconvection of gyrotactic microorganism. The novelty and purpose of this article is to develop a mathematical formulation for stagnation point flow along an extending or diminishing sheet including bioconvecting gyrotactic microorganisms. Buongiorno’s two-phase model is employed and spherical nanomaterials in a diluted concentration are mixed in base liquid. The fluid and heat transportation with concentration dependent characteristics is perceived to yield applicable outputs to enhance the thermal effectiveness of heat balancing equipments.

## Physical model and mathematical formulation

**Figure 1:**
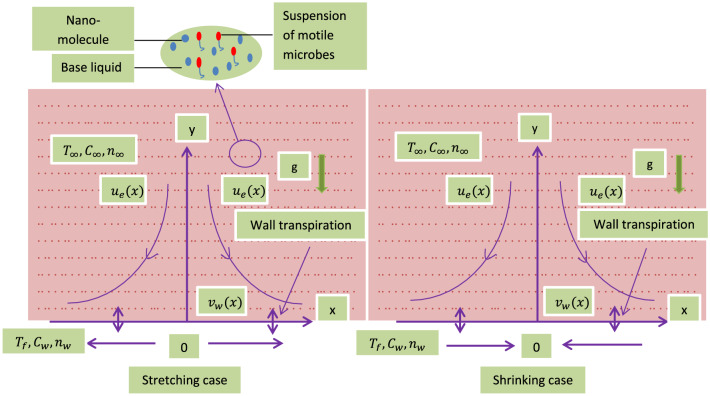
Flow geometry.

Consider two-dimensional flow of a tangent hyperbolic nanofluid encompassing self moving gyrotactic micro-organisms, a heat source, magnetic field, chemical reaction with activation energy and spherical nanoparticles in the region $y > 0$, caused by a permeable enlarging/contracting wall at y = 0 with a stagnation point at x = 0 (see Fig. 1). The wall temperature is $T_w$, uniform nanofluid concentration is $C_w$ as well as uniform concentration of motile microorganisms is $n_w$.

By modifying the framework of^52^ to encompass variable viscosity (see^52,53^), the regulating equations for conserving of mass, momentum, energy, concentration, and motile microorganism concentration can be stated as follows^13,52,54,55^:

**Continuity Equation:**

1 $$\begin{aligned} \frac{\partial u}{\partial x} + \frac{\partial v}{\partial y} = 0, \end{aligned}$$

**Momentum Equation:**

$$\begin{aligned} u\frac{\partial u}{\partial x} +v \frac{\partial u}{\partial y}= u_e \frac{du_e}{dx} +\left( \frac{1}{\rho _{\infty }} \right) \frac{\partial }{\partial y}[\mu (C)\frac{\partial u}{\partial y}]+v(1-\epsilon )\frac{\partial ^2 u}{\partial y^2} +\sqrt{2}v\varGamma \epsilon \frac{\partial u}{\partial y}.\frac{\partial ^2 u}{\partial y ^2}-\left( \frac{\sigma ^{*} B_o}{\rho }+\frac{v}{k_1}\right) (u-u_e) \end{aligned}$$

2 $$\begin{aligned} + \left( \frac{1}{\rho }\right) \left[ (1-C_\infty )\rho _\beta (T-T_\infty )\right] -(\rho _p-\rho _f)g(C-C_{\infty })-(n-n_{\infty })g\gamma (\rho _m-\rho )], \end{aligned}$$

**Energy Equation:**

3 $$\begin{aligned} u \frac{\partial T}{\partial x}+v \frac{\partial T}{\partial y}=\frac{1}{\rho _\infty c_p}\frac{\partial }{\partial y}\left[ k(C)\frac{\partial T}{\partial y}\right] +\tau \frac{\partial }{\partial y}\left[ D_B\left( C\right) (C-C_\infty )\right] \frac{\partial T}{\partial y}-\tau \frac{D_T}{T_\infty } \left( \frac{\partial T}{\partial y} \right) ^2 + Q_0 (T-T_\infty ), \end{aligned}$$

**Concentration Equation:**

4 $$\begin{aligned} u \frac{\partial C}{\partial x}+v \frac{\partial C}{\partial y}= \frac{\partial }{\partial y}\left[ D_B (C)\frac{\partial C}{\partial y}\right] + \frac{D_T}{T_{\infty }} \frac{\partial ^{2} T}{\partial y^{2}}-(Kr)^{2}(C-C_{\infty }) \left( \frac{T}{T_{\infty }} \right) ^{m^*}exp \left( \frac{-E_a}{k_2 T} \right) , \end{aligned}$$

**Motile density Equation:**

5 $$\begin{aligned} u \frac{\partial n}{\partial x}+v \frac{\partial n}{\partial y}= \frac{\partial }{\partial y}\left[ D_n (C)\frac{\partial n}{\partial y}\right] -\frac{bW_c}{\Delta C_w}\left[ \frac{\partial }{\partial y} \left( n\frac{\partial C}{\partial y} \right) \right] . \end{aligned}$$

along with the boundary conditions,

6 $$\begin{aligned}{}&u = u_{w}(x) = cx^{m}, v = v_{w}(x) = -\frac{m+1}{2} \sqrt{\frac{u_{e}(x) v_{\infty }}{x}} V_{0}, \ T = T_{f}, \ C = C_{w}, \ n = n_{w} \ \ at \ y=0,\nonumber \\&u \rightarrow \ u_{e} = ax^{m}, T \rightarrow \ T_{\infty }, C \rightarrow \ C_{\infty }, n \rightarrow \ n_{\infty }, \ \ as \ y \rightarrow \infty . \end{aligned}$$

It is preferable to introduce the non-dimensional features *f*, $\theta$, $\phi$ and $\chi$ (for stream function, heat, nanofluid volume fraction and microorganism density) in quest to convert the governing partial differential equations to ordinary differential form, similarity variable $\eta$ and other functions are given below:

7 $$\begin{aligned} \eta = y \sqrt{\frac{u_{e}(x)}{v_{\infty }} x}, \ \ \psi = \sqrt{u_{e} (x) v_{\infty }} xf(\eta ), \ \ \theta (\eta ) = \frac{T - T_{\infty }}{T_{f} - T_{\infty }}, \ \ \phi (\eta ) = \frac{C - C_{\infty }}{C_{w} - C_{\infty }}, \ \ \chi (\eta ) = \frac{n - n_{\infty }}{n_{w} - n_{\infty }}. \end{aligned}$$

The concentration dependent physical quantities are mentioned as under:

8 $$\begin{aligned}{}&\mu (C) = \mu _{\infty } \left[ 1 + c_1 (C - C_{\infty } ) \right] = \mu _{\infty } \left[ 1+ c_2 \phi (\eta ) \right] , \end{aligned}$$

9 $$\begin{aligned}{}&k(C) = k_{\infty } \left[ 1 + c_3 (C - C_{\infty } ) \right] = k_{\infty } \left[ 1+ c_4 \phi (\eta ) \right] , \end{aligned}$$

10 $$\begin{aligned}{}&D_B(C) = D_{B,{\infty }} \left[ 1 + c_5 (C - C_{\infty } ) \right] = D_{B,{\infty }} \left[ 1+ c_6 \phi (\eta ) \right] , \end{aligned}$$

11 $$\begin{aligned}{}&D_n(C) = D_{n,{\infty }} \left[ 1 + c_7 (C - C_{\infty } ) \right] = D_{n,{\infty }} \left[ 1+ c_8 \phi (\eta ) \right] . \end{aligned}$$

Equation (1) is satisfied. Equations (1)–(5) are transformed into a system of non-linear ODE’s using the Eqs. (7)–(11).

12 $$\begin{aligned}{}&\left[ ( 1+ c_2 \phi ) + ( 1-\epsilon )+ \frac{\epsilon }{\sqrt{2}}{\frac{2}{m+1}} Wef'' \right] f''' + c_2 \phi ' f'' +\frac{m+1}{2} \, ff'' -mf'^2 + (M+ Kp) (1-f') + \omega ( \theta - Nr \phi - Rb \chi ) + m = 0, \end{aligned}$$

13 $$\begin{aligned}{}&( 1+ c_4 \phi ) \theta ''+ Pr\frac{m+1}{2}f \theta ' + c_4\theta ' \phi '+Nb(1+2c_6\phi )\theta '\phi ' +Nt\theta '^2 +Q \theta = 0, \end{aligned}$$

14 $$\begin{aligned}{}&(1+ c_6 \phi ) \phi '' + Le \frac{m+1}{2}f\phi '+ c_6\phi '^2 + \frac{Nt}{Nb}\theta '' -LeA\phi (1+\delta \theta )^{m^*} \exp \left( \frac{-E}{1+\delta \theta } \right) = 0, \end{aligned}$$

15 $$\begin{aligned}{}&( 1+ c_8 \phi ) \chi '' + Sc \frac{m+1}{2}f\chi '+c_8 \phi ' \chi ' -Pe\left[ \phi '\chi '+\phi ''(\sigma +\chi )\right] = 0. \end{aligned}$$

The transformed boundary conditions Eq. (6) are:

16 $$\begin{aligned}{}&f(0) = V_0, \ f'(0) = \lambda , \ \theta (0)=1, \ \phi (0) = 1, \ \chi (0) = 1,\nonumber \\&f'(\infty ) \rightarrow 1, \ \theta (\infty ) \rightarrow 0, \ \phi (\infty ) \rightarrow 0, \ \chi (\infty ) \rightarrow 0. \end{aligned}$$

The non-dimensional parameters are described as: $We=\sqrt{\frac{a^3 \Gamma ^2(m+1)^2 x^{3m-1}}{\nu _{\infty }}}$, $M = \frac{\sigma B_0^{2}}{a \rho x^{m-1}}$, $Kp = \frac{v}{ak_1 x^{m-1}}$, $\omega = \frac{\beta g (1- C_{\infty }) ( T_f - T_{\infty })}{ \rho a^{2} x^{2m-1}}$, $Nr = \frac{ ( \rho _p - \rho )(C_w - C_{\infty })}{ \beta \rho (1 - C_{\infty }) (T_f - T_{\infty })}$, $Rb = \frac{\gamma ^* (n_w - n_{\infty })( \rho _m - \rho )}{ \beta \rho (1- C_{\infty }) (T_f - T_{\infty })}$, $A = \frac{(Kr)^2}{ax^{m-1}}$, $E = \frac{E_a}{K_2 T_{\infty }}$, $Pr = \frac{c_p \mu _{\infty }}{k_{\infty }} = \frac{v_{\infty }}{\alpha _{\infty }}$, $Q = \frac{Q_0}{a x^{m-1}}$, $Le = \frac{v_{\infty }}{D_{B, {\infty }}}$, $Nb = \frac{\tau D_B (C_w - C_{\infty })}{\alpha _{\infty }} = \frac{ \tau D_B \Delta C_w}{\alpha _{\infty }}$, $Nt = \frac{ \tau D_T (T_f - T_{\infty })}{\alpha _{\infty } T_{\infty }} = \frac{ \tau D_T \Delta T_f}{\alpha _{\infty } T_{\infty }}$, $Sc = \frac{v_{\infty }}{D_{n, \infty }}$, $Pe = \frac{bW_c}{D_{n, \infty }}$, $\delta = \frac{T_f - T_{\infty }}{T_{\infty }}$, $\Omega = \frac{n_{\infty }}{n_w - n_{\infty }} = \frac{n_{\infty }}{\Delta n_w}$, $\lambda = \frac{c}{a}$.

## Physical quantities

### Skin friction coefficient

The skin friction coefficient is calculated as follows:

$$\begin{aligned} Cf_x = \frac{\tau _w}{\rho u_e^{2}}, \end{aligned}$$

where, $\tau _w$ is described as the force exerted by a moving object and defined as:

$$\begin{aligned} \tau _w = \mu (C)\left[ (1- \epsilon ) \frac{\partial u}{\partial y} + \epsilon \Gamma \left( \frac{\partial u}{\partial y}\right) ^{2} \right] \ \ \ \ at \ \ y = 0, \end{aligned}$$

Hence,

$$\begin{aligned} Cf_x(Re_x)^{ \frac{1}{2}} = (1+c_2 \phi (0)) \left[ (1- \epsilon ) f''(0) + \frac{\epsilon }{m+1} We (f''(0))^{2} \right] . \end{aligned}$$

where,

$$\begin{aligned} Re_x = \frac{xu_e}{v_{\infty }} \ is \ the\ local \ Reynolds \ number. \end{aligned}$$

### Local Nusselt number

The mathematical expression for the relation of thermal transport efficiency is as follows:

$$\begin{aligned} Nu_x = \frac{xq_w}{k(C) (T_f - T_\infty )}, \end{aligned}$$

where, the surface heat transfer is symbolized by the notation $q_w$ and is stated as:

$$\begin{aligned} q_w = - k(C) \frac{\partial T}{\partial y} \ \ \ \ at \ \ y = 0, \end{aligned}$$

Using Eqs. (7) and (9), the above equation is reduced as:

$$\begin{aligned} Nu_x(Re_x)^{-1/2} = -\theta '(0). \end{aligned}$$

### Sherwood number

The mass transmission rate factor has the following mathematical interpretation:

$$\begin{aligned} Sh_x = \frac{xq_m}{D_B(C)(C_w - C_\infty )}, \end{aligned}$$

where, Surface mass flux is denoted by *qm*, which is expressed as:

$$\begin{aligned} q_m = -D_B(C) \frac{\partial C}{\partial y} \ \ \ \ at \ \ y = 0, \end{aligned}$$

Using Eqs. (7) and (10), the dimensionless form of above equation is:

$$\begin{aligned} Sh_x(Re_x)^{-1/2} = -\phi '(0). \end{aligned}$$

### Density number of micro-organisms

The Local density of miro-organisms is described as:

17 $$\begin{aligned} Nn_x = \frac{xq_w}{D_n(C)(n-n_{\infty })}, \end{aligned}$$

where $q_w$ identifies the flux of motile microbes and is delineated as:

18 $$\begin{aligned} P_w = - D_n(C) \frac{\partial n}{\partial y} \ \ \ \ at \ \ y = 0, \end{aligned}$$

Using Eqs. (7) and (11), the non-dimensional form of equation is:

$$\begin{aligned} Nn_x(Re_x)^{ \frac{-1}{2}} = - \chi '(0). \end{aligned}$$

## Solution procedure

This portion comprises results obtained from the non-linearly related standard differential Eqs. (12)–(15) with boundary Eq. (16), to be evaluated using the RK-4 method. The higher order derivatives in these equations are reduced to first order to develop coding of the numerical procedure. We let,

$$f_1'$$ = $$f_2$$

$$f_2'$$ = $$f_3$$

$$ f_3' = \frac{-1}{ \left[ (1+ c_2 f_6)+ (1- \epsilon )+{\frac{\epsilon }{\sqrt{2}} \frac{2}{m+1}}Wef_3 \right] } \left[ c_2 f_3f_7 + \frac{m+1}{2} \, f_1 f_3 - mf_2^{2} +(M+Kp)(1-f_2) + \omega (f_4- Nrf_6-Rbf_8 ) +m \right] $$

$$f_4'$$ = $$f_5$$

$$f_5'=\frac{-1}{ (1+ c_4 f_6)}\left[ Pr \frac{m+1}{2} \, ff_5 + c_4 f_5 f_7 + Nb(1+2c_6f_6) \, f_5f_7 + Ntf_5^{2} +Qf_4 \right] $$

$$f_6'$$ = $$f_7$$

$$f_7' = \frac{-1}{(1+ c_6 f_6)} \left[ Le \frac{m+1}{2} \,  f f_7+ c_6f_7^{2} + \frac{Nt}{Nb} \, f'_5 -LeAf_6(1+ \delta f_4)^{m^*} \exp \left( \frac{-E}{1+ \delta f_4}\right) \right] $$

$$f_8'$$ = $$f_9$$

$$f_9' =\frac{-1}{ \left( 1 + c_8 f_6 \right) } \left[ Sc \frac{m+1}{2} \, f_1 f_9 + c_8 f_7f_9 -Pe\left[ f_7f_9 +f'_7(\Omega +f_8)\right] \right] $$

along with the boundary conditions: $f_1 = S, \ f_2 = \lambda , \ f_4 = 1, \ f_6 = 1, \ f_8 = 1 \ \ at \ \ \eta = 0$,

$$f_2 \rightarrow 1, \ f_4 \rightarrow 0, \ f_6 \rightarrow 0, \ f_8 \rightarrow 0 \ as \ \eta \rightarrow \infty $$.

## Results and discussion

This segment presents mass and heat transport features of a tangent hyperbolic fluid across a stratching/shrinking sheet comprising heat formation, chemical change with activation energy as well as bioconvection. The consequences of influential factors such as magnetic parameter *M*, Weissenberg number *We*, material power law index $\epsilon$, viscosity parameter $c_2$, suction/injuction parameter $V_o$, porosity paramter *Kp*, mixed convection parameter $\omega$, Rayleigh number *Rb*, buoyancy ratio parameter *Nr*, thermal conductivity parameter $c_4$, Prandtl number *Pr*, Brownian Motion parameter *Nb*, mass diffusivity parameter $c_6$, thermophoresis parameter *Nt*, heat source parameter *Q*, micro-organisms species diffusivity parameter $c_8$, Activation energy *E*, Chemical reaction parameter *A*, Lewis number *Le*, bioconvection constant $\Omega$, Schmidt number *Sc* and Peclet number *Pe* on velocity pattern, temperature dispersion, concentration profile and motile concentration distribution are displayed graphically. Physical factors like skin friction, Nusselt number, Sherwood number and motile density factor are expressed in tabulated form. Table 1 entities $f''(0)$, $\theta '(0)$ and $\phi '(0)$ as evaluated and those presented previously by Alsenafi et al.^52^ and Zaimi et al.^56^, respectively. The comparison was carried out while neglecting the presence of gyrotactic microorganisms (by omitting Eq. (15) and allocating $V_0$ = 0 and $\lambda$ = 1 in the boundary limitations (16)).

**Table 1:** The comparative outputs.

	Alsenafi et al.^52^	Zaimi et al.^56^	Present results
$f''(0)$	0	0	0
$-\theta '(0)$	0.476745	0.476737	0.4768
$-\phi '(0)$	1.045230	1.045154	1.0453

Tables 2 and 3 reveal the effects of diverse parameters on the Skin friction factor $-f''(0)$ and Nusselt number $-\theta '(0)$. Table 2 shows that as $c_2$, $\epsilon$, *We*, *M*, *Kp*, $\omega$, *Nr* and *Rb* increase, the skin friction coefficient $-f''(0)$ reduces. Table 3 illustrates that as $c_4$, *Nb*, *Nt* and *Q* are applied, the Nusselt number magnitude reduces considerably, but it rises as *Pr* is enhanced. Tables 4 and 5 show the effect of controlling parameters on Sherwood factor $-\phi '(0)$ as well as the motile density number $-\chi '(0)$. Table 4 displays that the local Sherwood number $-\phi '(0)$ is directly enhanced with *Le*, *Nb*, *A* and *Nt*, but it reduces as *E* and $c_6$ are evolved. As per Table 5, the motile concentration factor $-\chi '(0)$ is obviously improved by *Sc*, *Pe* and $\Omega$, although it diminishes against $c_8$.

**Figure 2:**
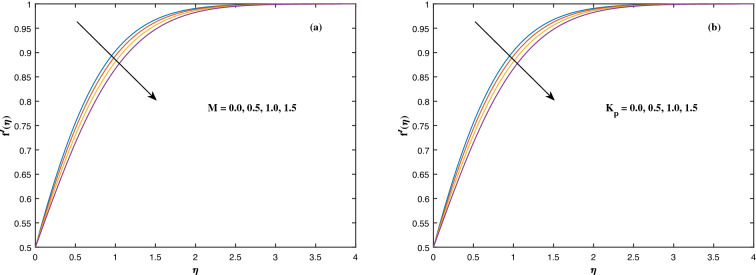
Fluctuation in $f'(\eta )$ with (**a**) *M* and (**b**) $K_p$.

**Figure 3:**
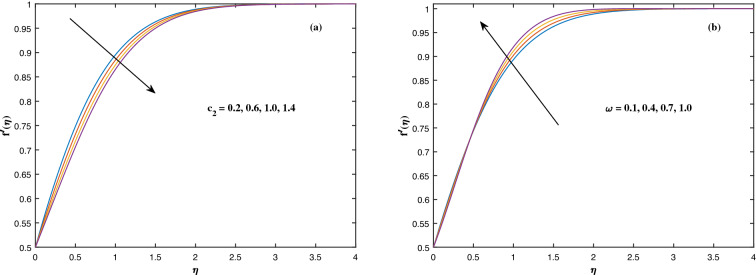
Fluctuation in $f'(\eta )$ with (**a**) $c_2$ and (**b**) $\omega$.

**Figure 4:**
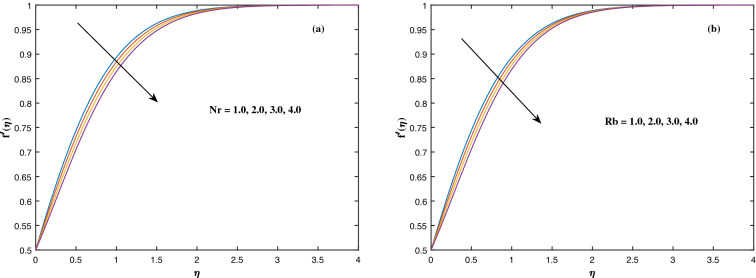
Fluctuation in $f'(\eta )$ with (**a**) *Nr* and (**b**) *Rb*.

**Figure 5:**
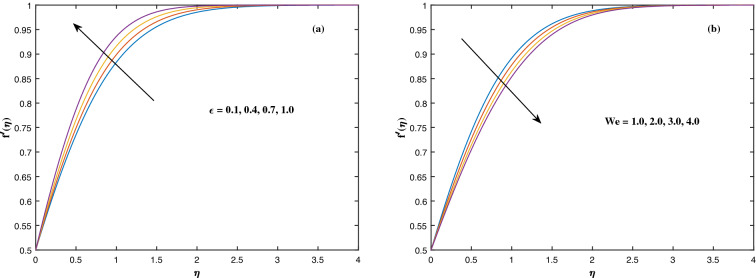
Fluctuation in $f'(\eta )$ with (**a**) $\epsilon$ and (**b**) *We*.

**Figure 6:**
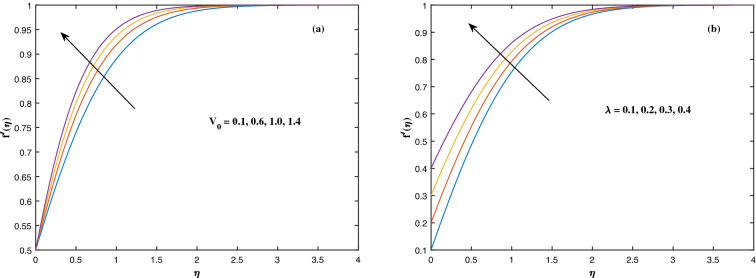
Fluctuation in $f'(\eta )$ with (**a**) $V_0$ and (**b**) $\lambda$.

**Figure 7:**
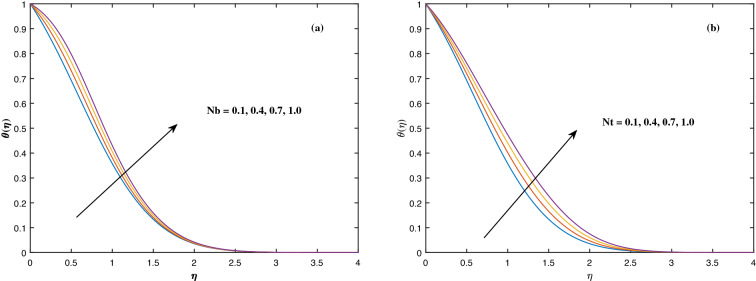
Fluctuation in $\theta (\eta )$ with (**a**) *Nb* and (**b**) *Nt*.

**Figure 8:**
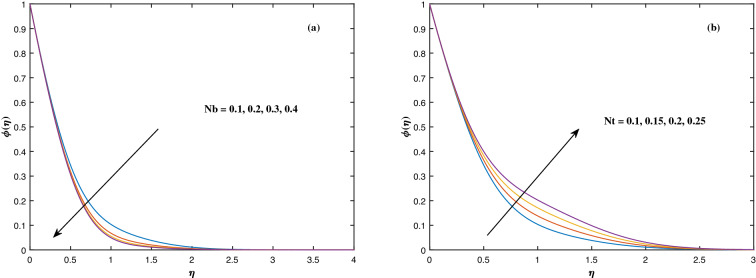
Fluctuation in $\phi (\eta )$ with (**a**) *Nb* and (**b**) *Nt*.

**Figure 9:**
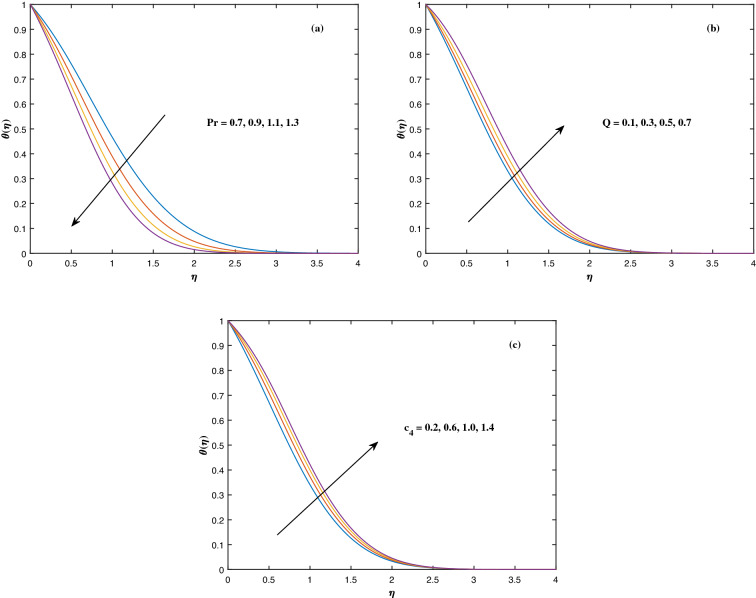
Fluctuation in $\theta (\eta )$ with (**a**) *Pr*, (**b**) *Q* and (**c**) $c_4$.

**Figure 10:**
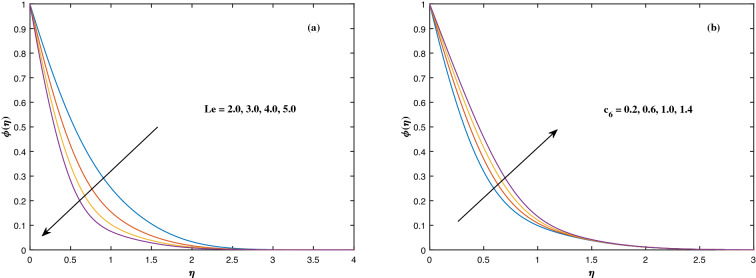
Fluctuation in $\phi (\eta )$ with (**a**) *Le* and (**b**) $c_6$.

**Figure 11:**
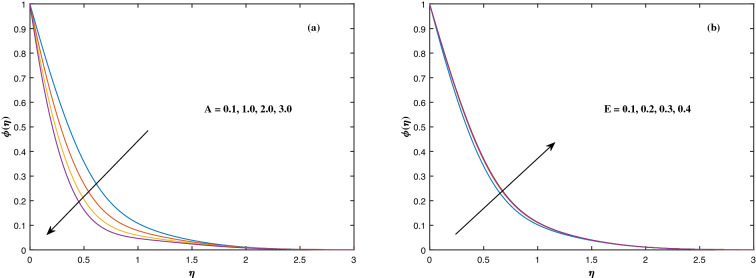
Fluctuation in $\phi (\eta )$ with (**a**) *A* and (**b**) *E*.

**Figure 12:**
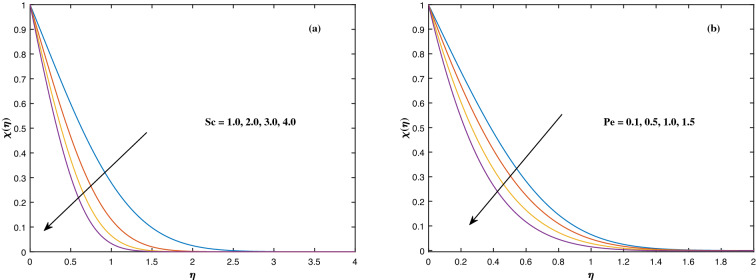
Fluctuation in $\chi (\eta )$ with (**a**) *Sc* and (**b**) *Pe*.

**Figure 13:**
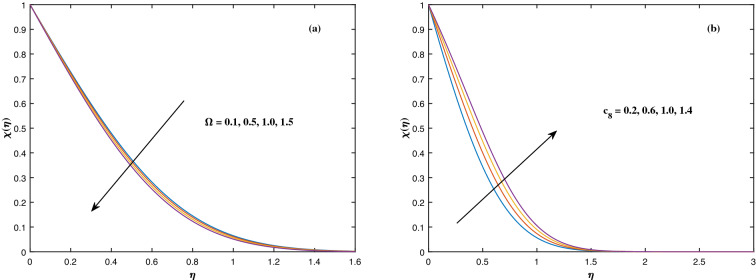
Fluctuation in $\chi (\eta )$ with (**a**) $\Omega$ and (**b**) $c_8$.

The suitable ranges of parameters are taken as $0.0 \le M \le 1.5$, $0.0 \le K_P \le 1.5$, $0.2 \le c_2 \le 1.4$, $0.1 \le \omega \le 1.0$, $1.0 \le Nr \le 4.0$, $1.0 \le Rb \le 4.0$, $0.1 \le \epsilon \le 1.0$, $1.0 \le We \le 4.0$, $0.1 \le V_0 \le 1.4$, $0.1 \le \lambda \le 0.4$, $0.1 \le Nb \le 1.0$, $0.1 \le Nt \le 1.0$, $0.7 \le Pr \le 1.3$, $0.1 \le Q \le 0.7$, $0.2 \le c_4 \le 1.4$, $2.0 \le Le \le 5.0$, $0.2 \le c_6 \le 1.4$, $0.1 \le A \le 3.0$, $0.1 \le E \le 0.4$, $1.0 \le Sc \le 4.0$, $0.1 \le Pe \le 1.5$, $0.1 \le \Omega \le 1.5$ and $0.2 \le c_8 \le 1.4$. Figure 2 depicts how the magnetic parameter *M* and the porosity parameter *Kp* affect the velocity distribution $f'(\eta )$. It is observed that raising the magnitude of *M* reduces the velocity field of the liquid. The magnetic factor relates to the relationship between electro-magnetic force and viscosity. Lorentz force originated in the flow as a consequence of the interaction of electric and magnetic field, and there is a significant link between Lorentz force and magnetic flux, so that as magnetic flux rises, Lorentz force grows, producing restricting force to strengthen and velocity to reduce substantially. The velocity is observed to decline as the value of *Kp* rises. It is simply related to resistance of porous media, $K_P$ being reciprocal to permeability is capable to that efficiently inhibit the movement of liquid particles. The consequences of nanofluid viscosity $c_2$ as well as mixed convection parameter $\omega$ on velocity profile $f'(\eta )$ are visualized in Fig. 3. The velocity distribution tends to decrease as the values of $c_2$ rise. The velocity significantly increases as the value of $\omega$ begins to rise. Figure 4 illustrates the effect of the buoyancy ratio variable *Nr* and the Rayleigh number *Rb* on the velocity distribution $f'(\eta )$. The velocity of the liquid reduces as the quantities *Nr* and *Rb* enhance. It is basically summarized as the existence of buoyant forces resulting in a reduction in velocity. Greater rates of natural and forced convection enhance heat transmission lower rates for a large flow of nanoparticles. The impacts of the power law index $\epsilon$ as well as the Weissenberg number *We* upon the velocity pattern are displayed in Fig. 5. As the value of $\epsilon$ amplifies, the velocity profile improves in an expanding trend. But, when the Weissenberg number *We* boosts, the velocity of the fluid declines. The Weissenberg factor is, in reality, a fluid relaxing time/viscous force. Relaxation time might be reduced as *We* get stronger. An extensive relaxation time allows the liquid to become thicker, offering extra restrictions to fluid flow. As an outcome, the fluid experiences a shear-thinning into shear-thickening transformation. Figure 6 exhibits how the stretching/shrinking variable $\lambda$ and the wall transpiration factor $V_0$ affect nanofluid velocity. It is evidenced that when the value of $\lambda$ improves, the thickness of the velocity boundary layer rises, and therefore the flow velocity enhances. It reveals that boosting the quantity $V_0$ strengthens the velocity of the liquid. Figures 7 and 8 demonstrate the influence of the Brownian motion parameter *Nb* as well as the thermophoresis variable *Nt* on the temperature profile $\theta (\eta )$ and concentration distribution $\phi (\eta )$, respectively. Figure 7 displays how temperature trends rise as *Nb* and *Nt* quantities are varied. The temperature is improved as the Brownian motion parameter is enhanced. The thermophoresis trend, on the other hand, accumulates the motion of movable nanomaterials into the cold zone, to raise temperature distribution. Figure 8 displays a concentration distribution that is diminished by modifying *Nb* values. The concentration variation improves as *Nt* enhances the optimum variability. Figure 9 scrutinizes the influence of the Prandtl number *Pr*, heat source factor *Q* and thermal conductivity variable $c_4$ on the temperature profile $\theta (\eta )$. Enhancing the Prandtl number *Pr* reduces the density of the thermal boundary layer. Actually, as the Prandtl number *Pr* rises, heat conductivity diminishes. The variation of temperature is defined as a relation of the heat source variable *Q*. As an outcome, the temperature variance in liquid $\theta (\eta )$ is amplified. It is also noted that when the quantity $c_4$ grows, so does the temperature pattern. Figure 10 exhibits the response of the nanoparticle volume fraction $\phi (\eta )$ to alterations in the Lewis factor *Le* as well as the mass diffusivity of the nanoparticles $c_6$. The inputs demonstrated that expanding the amounts of *Le* reduces concentration intensity since the Lewis number hinders fluid flow, whereas increasing the amounts of $c_6$ boosts nanoparticle concentration. Figure 11 depicts the association between the chemical change parameter *A* and the activation energy *E* upon the concentration pattern $\phi (\eta )$. The concentration of nanoparticles diminishes as the quantity of the chemical change variable *A* grows. However, rising *E* values boosts the concentration of nanomaterials. This perspective is based on the assumption that because of the lower heat and strong activation, the strength of the reaction is minimized, which improves fluid concentration. The consequences of the Schmidt quantity *Sc* as well as the Peclet number *Pe* upon motile density dispersion $\chi (\eta )$ can be seen in Fig. 12. The Schmidt factor *Sc* is associated with the species distribution stream in reciprocal way. As a response, boosting this parameter induces the microorganism concentration profile to diminish. Stronger *Pe* causes to reduce microorganism diffusivity. Figure 13 displays how the bioconvection variable $\Omega$ and the microbe species conductivity variable $c_8$ influence the motile density distribution $\chi (\eta )$. Because $\Omega$ is a key component in liquid motility, higher inputs of $\Omega$ is capable to lower the concentration of motile microbes. While the microorganism species diffusivity parameter $c_8$ is raised, the quantitative values of microorganism density rise notably.

**Table 2:** Results for skin friction factor $-f''(0)$.

$c_2$	$\epsilon$	*We*	*M*	$K_p$	$\omega$	*Nr*	*Rb*	$-f''(0)$
0.2	0.3	2.0	0.5	0.5	0.1	1.0	1.0	0.2102
0.4								0.0981
0.6								0.0046
0.4	0.1							0.1548
	0.2							0.1267
	0.3							0.0981
	0.3	1.0						0.1169
		2.0						0.0981
		3.0						0.0795
		2.0	0.5					0.0981
			1.5					0.0874
			1.5					0.0754
			0.5	0.1				0.1061
				0.3				0.1022
				0.5				0.0981
				0.5	0.1			0.0981
					0.2			0.0947
					0.3			0.0912
					0.1	0.1		0.1042
						0.5		0.1015
						1.0		0.0981
						1.0	0.1	0.1043
							0.5	0.1016
							1.0	0.0981

**Table 3:** Results for Nusselt number $-\theta '(0)$.

$c_4$	*Nb*	*Nt*	*Q*	$-\theta '(0)$
0.2	0.1	0.1	0.3	0.5620
0.4				0.5030
0.6				0.4564
0.4	0.1			0.5030
	0.2			0.4532
	0.3			0.4085
	0.1	0.1		0.5030
		0.2		0.4835
		0.3		0.4649
		0.1	0.1	0.5864
			0.2	0.5455
			0.3	0.5030

**Table 4:** Results for Sherwood number $-\phi '(0)$.

$c_6$	*Le*	*Nt*	*Nb*	*A*	*E*	$-\phi '(0)$
0.2	4.0	0.1	0.1	0.2	0.3	1.8291
0.4						1.6407
0.6						1.4967
0.4	3.0					1.3801
	4.0					1.6407
	5.0					1.8753
	4.0	0.1				1.6407
		0.2				1.6464
		0.3				1.6646
		0.1	0.1			1.6407
			0.2			1.6588
			0.3			1.6636
			0.1	0.1		1.5705
				0.2		1.6407
				0.3		1.7084
				0.2	0.1	1.6684
					0.2	1.6540
					0.3	1.6407

**Table 5:** Results for motile density number $-\chi '(0)$.

$c_8$	*Sc*	*Pe*	$\Omega$	Non-linear case $-\chi '(0)$
0.2	3.0	0.1	1.0	1.5537
0.4				1.3860
0.6				1.2557
0.4	3.0			1.3860
	4.0			1.6171
	5.0			1.8311
	3.0	0.1		1.3860
		0.2		1.4828
		0.3		1.5806
		0.1	0.1	1.3860
			0.2	1.3931
			0.3	1.4003

## Conclusions

In an attempt to assess newly emerging bio-inspired nanofluid films enveloping manufacturing mechanisms, a mathematical template besides stagnation point stream toward an enlarging or dwindling layer of Tangent hyperbolic liquid nano-biofilm encompassing spherical nano-particles, chemical processes with activation energy, and gyrotactic microorganisms has been elaborated. Analytical correlations were applied to variable fluid transport properties (viscosity, thermal conductivity, nanoparticle mass diffusion coefficient) and also to microorganisms (species diffusivity). The following are the significant findings from the most current computations:

- With the modification of magnetic parameter, porosity factor, viscosity parameter, Rayleigh number, buoyancy ratio parameter and Weissenberg number, a diminishing velocity pattern was found that is considerably advanced if the mixed convection parameter, stretching/shrinking parameter, material power law index and wall transpiration parameter are analyzed.
- Brownian motion, thermal conductivity, heat generation as well as thermophoresis factors all strengthen the temperature distribution, however the Prandtl number lowers the temperature profile.
- The nano-particle concentration profile is enhanced as the nano-particle mass conductivity variable, activation energy as well as the thermophoresis variable boost, but it gradually decreases as the Lewis number, chemical reaction rate and Brownian motion factor rise.
- Microorganism density improves significantly when the microbe diffusivity factor grows, but reduces considerably as the Schmidt number, Peclet number and bioconvection constant grow.
- While the amounts of the parameters thermal conductivity factor, Brownian motion factor, thermophoresis variable as well as heat source parameter improved, the heat transfer rate declined while improving as Prdantl number enhanced.
- The Sherwood number reduces when the variables activation energy parameter and nano-particle mass conductivity factor grow, whereas it boosts as the variables Lewis number, Brownian motion factor, chemical reaction factor and thermophoresis factor expand.
- The motile density number diminishes as the microorganism species conductivity parameter rises, but it accelerates for Peclet number, Schmidt number and bioconvection factor.

## Data Availability

Fully documented templates are available in the elsarticle package on CTAN (https://ctan.org/tex-archive/macros/latex/contrib/elsarticle).

## Latin symbols

*u*, *v*: Nanofluid velocity components
(*x*, *y*): Cartesian Coordinates
$u_w(x)$: Stretching/shrinking velocity
$u_e(x)$: Ambient fluid velocity
$v_w(x)$: Wall transpiration (suction/blowing) velocity
$B_0$: Magnetic field strength
*C*: Concentration of nanoparticles
*T*: Temperature of nanoparticles
*n*: Density of micro-organisms
$k_1$: Permeability of porous medium
*k*(*C*): Variable thermal conductivity
$k_{\infty }$: Constant thermal conductivity
$c_p$: Specific heat at constant pressure
*g*: Gravitational accerlation
*c*: Constant in stretching/shrinking velocity
$$c_2$$: Viscosity parameter
$c_4$: Thermal conductivity parameter
$c_6$: Nanoparticle mass diffusivity
$c_{8}$: Micro-organisms species diffusivity
$D_B (C)$: Variable Brownian diffusion coefficient
$D_T$: Thermophoresis diffusion coefficient
$D_n (C)$: Variable diffusivity of micro-organisms
$D_{B, \infty }$: Constant nano-particle mass diffusivity
$D_{n, \infty }$: Constant micro-organisms diffusivity
$T_{\infty }$: Uniform temperature in the free stream
$C_{\infty }$: Uniform nanofluid concentration in free stream
$n_{\infty }$: Uniform density of microorganisms in free stream
$Q_0$: Heat source/sink coefficient
*M*: Magnetic field parameter
*Kp*: Porosity parameter
*Pr*: Prandtl number
*Nb*: Brownian motion parameter
*Nt*: Thermophoresis parameter
*Sc*: Schmidt number
*Q*: Heat source parameter
*Le*: Lewis number
*Pe*: Peclet number
*Nr*: Buoyancy ratio parameter
*Rb*: Rayleigh number
$W_c$: Maximum cell swimming speed
$W_e$: Local Weissenberg number
$T_w$: Uniform temperature at the sheet surface
$C_w$: Uniform nanoparticles concentration at sheet surface
$n_w$: Uniform density of micro-organisms at sheet surface
*f*: Dimensionless stream function
*A*: Chemical reaction rate
*a*: Constant in the ambient fluid velocity
*b*: Chemotaxis constant
$V_0$: Wall transpiration parameter

## Greek symbols

$\alpha _{\infty }$: Uniform thermal diffusivity
$\mu _{\infty }$: Constant dynamic viscosity
$\mu (C)$: Variable dynamic viscosity
$$\nu $$: Kinematic viscosity
$$\Omega $$: Bioconvection constant
$$\sigma ^{*}$$: Electrical conductivity
$\rho$: Density of fluid
$\rho _{\infty }$: Constant fluid density
$\gamma$: Average volume of micro-organisms
$\omega$: Mixed convection parameter
$\lambda$: Stretching/shrinking parameter
$\Gamma$: Material time constant
$$\delta $$: Temperature difference
$\psi$: Dimensionless stream function
$\eta$: Dimensionless transverse coordinate
$\theta$: Dimensionless temperature function
$\phi$: Dimensionless concentration of nanoparticles
$\chi$: Dimensionless density of micro-organisms
